# Letter from Paris

**Published:** 1868-09

**Authors:** D'Oyley Evans


### CORRESPONDENCE.

### ARTICLE VI.

### *Letter from Paris.*

2 Place de la Trinite,

Paris, *June* 7, 1868.

Dear Doctor:—

In my letter, dated July 10th, 1867, I mentioned a new method of introducing medicine into the animal economy through the nose. Lately an article has been published in the

*Journal des Connaissances Medicales*, written by Dr. Raimbert, apropos to the above system, and his experience having been very successful in various affections of the head, I hope that we may be able to apply it in cases of neuralgic toothache, so frequently met with in Dental practice. The pituitary membrane, Dr. R. observes, has been hitherto quite neglected by physicians as an organ of absorption, the only use made of it having been hitherto restricted to irritating it for the purpose of exciting stranulation.

Dr. Rainbert however, having remarked that a powder composed of calomel, red precipitate, and sugar candy, and administered like snuff in a case of ozena, had caused salivation, thought it highly probable that the nose might become a vehicle for other substances too. Actuated by this idea he prescribed, a powder composed, of one grain of marsh-mallow and five centigms. of morphine, in the case of a gentleman, aged 30, who while laboring under a severe attack of influenza accompanied with a coryza, experienced a violent pain in the left sub-orbital nerve. The pain had lasted twenty-four hours, had prevented his night's rest, and increased by fits. The above powder was taken by the patient in pinches like snuff, at the rate of one pinch every second or third hour. In the evening the pain abated, the patient slept well the whole night, and on the following day every trace of pain had disappeared.

In a similar manner Dr. Raimbert cured a very violent headache by a mixture of 2 gms. of finely porphyrized sugar, and 5 centigms. of hydrochlorate of morphine. Two cases of neuralgic toothache I have treated with success, one patient aged 62 the other a young man of 19 years.

The former was considerably relieved, and the latter perfectly cured, by 2 gms of sugar and 10 centig. of hydrochloate of morphine. I shall be gratified if others of our profession will experiment and report their success.

I copied from the *London Lancet of January* 1868, an interesting chronological history of painless surgical operations during the anæsthetic state, induced by the inhalation of narcotic and stimulating vapours; and thinking that it may interest you, I insert it. "The first surgical operation during an anæsthetic condition, induced by the inhalation of the fumes of Rum, was the reduction of a dislocation of a hip joint, of a negro "Bob," Louisiana, by Dr. Collyer, December 1839.

Extraction of a tooth for Miss Mary Allen during an insensible condition, induced by the inhalation of ether combined with the fumes of poppy-seeds. Philadelphia, by Dr. Collyer, November 1842.

Insensibility produced by the inhalation of protoxide of nitrogen. Hartford, Connecticut, Horace Wells, 1845.

Publication in Boston Medical Journal that ether combined with opium would produce the anæsthetic state. Boston, by Dr. Smilie, June 1846.

Administration of ether by Drs. Morton and Jackson. Boston, September 1846.

Inhalation of Chloroform. Edinburgh, by D. Simpson, 1854.

Amylene. London, by Dr. Snow, 1857.

Bichloride of Methylene. London, by Dr. Richardson 1867."

Ever yours respectfully
D'OYLEY EVANS.

---

